# Structural Covariance Network Disruption and Functional Compensation in Parkinson’s Disease

**DOI:** 10.3389/fnagi.2020.00199

**Published:** 2020-07-02

**Authors:** Cheng Zhou, Ting Gao, Tao Guo, Jingjing Wu, Xiaojun Guan, Weiwen Zhou, Peiyu Huang, Min Xuan, Quanquan Gu, Xiaojun Xu, Shunren Xia, Dexing Kong, Jian Wu, Minming Zhang

**Affiliations:** ^1^Department of Radiology, The Second Affiliated Hospital, Zhejiang University School of Medicine, Hangzhou, China; ^2^Department of Neurology, The Second Affiliated Hospital, Zhejiang University School of Medicine, Hangzhou, China; ^3^Department of Radiation Oncology, Sir Run Run Shaw Hospital, Zhejiang University School of Medicine, Hangzhou, China; ^4^Zhejiang University City College, Hangzhou, China; ^5^School of Mathematical Sciences, Zhejiang University, Hangzhou, China; ^6^AdvanCed Computing aNd SysTem Laboratory, College of Computer Science and Technology, Zhejiang University, Hangzhou, China

**Keywords:** Parkinson’s disease, neural network, blood oxygen level-dependent, magnetic resonance imaging, independent component analysis

## Abstract

**Purpose**: To investigate the structural covariance network disruption in Parkinson’s disease (PD), and explore the functional alterations of disrupted structural covariance network.

**Methods**: A cohort of 100 PD patients and 70 healthy participants underwent structural and functional magnetic resonance scanning. Independent component analysis (ICA) was applied separately to both deformation-based morphometry (DBM) maps and functional maps with the same calculating parameters (both decomposed into 20 independent components (ICs) and computed 20 times the Infomax algorithm in ICASSO). Disrupted structural covariance network in PD patients was identified, and then, we performed goodness of fit analysis to obtain the functional network that showed the highest spatial overlap with it. We investigated the relationship between structural covariance network and functional network alterations. Finally, to further understand the structural and functional alterations over time, we performed a longitudinal subgroup analysis (51 patients were followed up for 2 years) with the same procedures.

**Results**: In a cross-sectional analysis, PD patients showed decreased structural covariance between anterior and posterior cingulate subnetworks. The functional components showed best overlap with anterior and posterior cingulate structural subnetworks were selected as anterior and posterior cingulate functional subnetworks. The functional connectivity between them was significantly increased [assessed by Functional Network Connectivity (FNC) toolbox]; and the increased functional connectivity was negatively correlated with cingulate structural covariance network integrity. Longitudinal subgroup analysis showed cingulate structural covariance network disruption was worse at follow-up, while the functional connectivity between anterior and posterior cingulate network was increased at baseline and decreased at follow-up.

**Conclusion**: This study indicated that the cingulate structural covariance network displayed a high susceptibility in PD patients. This study indicated that the cingulate structural covariance network displayed a high susceptibility in PD patients. Considering that disrupted structural covariance network coexisted with enhanced/remained functional activity during disease development, enhanced functional activity underlying the disrupted cingulate structural covariance network might represent a temporal compensation for maintaining clinical performance.

## Introduction

Parkinson’s disease (PD) is the second most common neurodegenerative disease, which is characterized by extensive Lewy body deposition. The pathological substance contributes to the degradation of the basal ganglia as well as the cerebral cortex, and thereby a variety of motor and non-motor symptoms are manifested in PD patients (Braak et al., 2004). With the deepening of the understanding, researchers believe that these clinical symptoms of PD are not caused by isolated brain lesions alone (Jellinger, 2012). The coexistence of multiple-region degeneration in a specific pattern, network disruption, plays an essential role in the development of PD (Seeley et al., 2009; Zeighami et al., 2015; de Schipper et al., 2017). Thus, exploring the change of brain network in PD patients can shed light on the neurodegenerative process and provide potentially effective therapeutic targets.

Structural change of PD can be reflected by noninvasive *in vivo* magnetic resonance imaging (MRI) technology. But most of the PD related structural MRI studies focused on the alteration on voxel-level or ROI-level. Although the atrophy of multiple brain regions such as basal ganglia, frontal, and parietal cortices was found (Lyoo et al., 2011; Pan et al., 2012; Mak et al., 2015), we still know less about the structural covariance network alteration in PD patients. Compared with the voxel-based and ROI-based analyses, the network-based approach can effectively identify specific networks with a preferential vulnerability to PD related pathobiology (Xu et al., 2009; Hafkemeijer et al., 2014, 2016). This approach is used to evaluate the network integrity which provides additional information on brain alterations. A low integrity score means a severe disruption of the network (Xu et al., 2009; Hafkemeijer et al., 2014, 2016; de Schipper et al., 2017, 2018). Preliminary evidence showed that the integrity of precentral gyri, paracingulate gyri, and parietal gyri covariance networks had higher classification performance for PD, and the disruption of cingulate covariance network was closely related to the non-motor symptom of PD (de Schipper et al., 2017; Lee et al., 2018). However, yet a consensus of structural covariance network alteration in PD patients was not reached currently. More importantly, the functional underpinning behind the altered structural covariance network is still unknown.

The structural covariance network showed high spatial overlap with classical functional networks gave us an insight that the structural covariance network possibly has related functional underpinning (Zeighami et al., 2015; de Schipper et al., 2017). It is meaningful to explore the relationship between structural covariance network and functional network alterations which could help to understand the brain functional reorganization in parkinsonian status (Gottlich et al., 2013; Baggio et al., 2015). Moreover, longitudinal observation of structural covariance network and functional network changes could provide a more comprehensive insight about disease progression.

Therefore, the goal of the present study was to explore the structural covariance network alteration and its association with the corresponding functional network in PD patients. We evaluated the structural covariance network integrity by combining deformation-based morphometry (DBM) with independent component analysis (ICA). Next, we assessed the alteration of the functional network that overlapped with the disrupted structural network. Then, we assessed the relationship between altered structural covariance network and functional network. Finally, a longitudinal analysis was conducted to evaluate the change of structural covariance network and functional network further. We hypothesized that network-based analysis could find out the fragile structural covariance network of PD patients, and disclose the functional modulation behind it.

## Materials and Methods

### Participants

**Figure 1:**
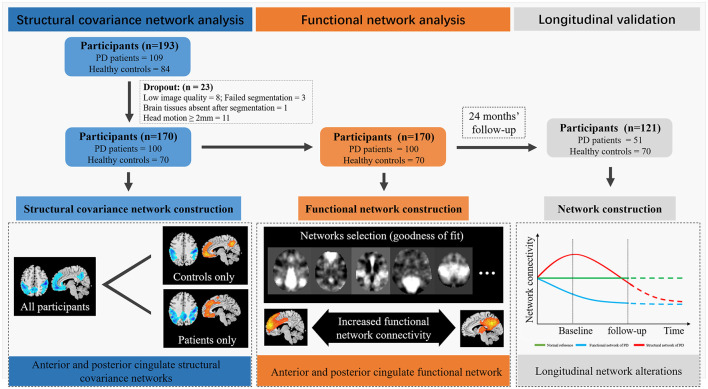
Flaw chart. The blue part of this image depicted the participant’s screening workflow and the results of structural covariance network analysis. Parkinson’s disease (PD) patients showed decreased structural covariance between anterior and posterior cingulate subnetworks. The orange part of this image depicted the flow and results of functional network analysis. The goodness of fit analysis was used to seek the functional network showed high spatial overlap with cingulate structural covariance network. Functional Network Connectivity (FNC) analysis showed increased connectivity between anterior and posterior cingulate network. The gray part of this image depicted the results of longitudinal validation. PD patients showed increased cingulate structural network disruption during follow-up, while the FNC increased first and decreased at last. Red curve, functional connectivity change; Blue curve, structural integrity change; Green-line, healthy control, Black-line, the two time-points in our data.

PD was diagnosed according to the United Kingdom Parkinson’s disease Society Brain Bank criteria by an experienced neurologist (Hughes et al., 1992). Subjects with a history of cerebrovascular disorders, head injury, neurological surgery, intracranial mass, or other neurological and psychiatric diseases were excluded from this study. For PD patients having anti-parkinsonian drugs, clinical assessments, and MRI scans were performed on drug-off status (>12 h). As shown in Figure 1, 109 PD patients and 84 age-matched healthy controls were initially enrolled. Twenty-three subjects were excluded because of the low quality of structural images or excessive head motion (transformation over 2 mm or/and rotation over 2 degrees) in functional images. As a result, a total of 170 subjects (100 PD patients and 70 healthy controls) were included in the structural covariance network and functional network analysis. From the original sample, 51 PD patients returned for longitudinal imaging and clinical assessments ~24 months after baseline. Healthy controls have no follow-up imaging and clinical assessment.

This study was approved by the Medical Ethics Committee of the Second Affiliated Hospital of Zhejiang University School of Medicine and all participants signed the informed consent forms following the Declaration of Helsinki. PD patients were recruited from the Department of Neurology of the Second Affiliated Hospital of Zhejiang University School of Medicine. Healthy controls were recruited from the communities.

### Clinical Assessment

Demographic information including age, sex, education, and disease duration was recorded. Clinical assessments including Unified Parkinson’s Disease Rating Scale (UPDRS), Hoehn-Yahr (H-Y) stage, and the Parkinson’s Disease Questionnaire-39 questions (PDQ-39) were evaluated in PD patients. The cognitive status of patients and healthy controls were evaluated using the Mini-Mental State Examination (MMSE).

### MRI Scanning and Data Processing

All subjects were scanned on a 3.0 Tesla MRI scanner (GE Discovery 750). The three dimensions T1 weighted (3D T1) images were acquired using a Fast Spoiled Gradient Recalled sequence: echo time = 3.036 ms; repetition time = 7.336 ms; inversion time = 450 ms; flip angle = 11°; field of view = 260 × 260 mm^2^; matrix = 256 × 256; slice thickness = 1.2 mm; 196 sagittal slices. Rs-fMRI images were acquired using Gradient Recalled Echo—Echo Planar Imaging sequence: echo time = 30 ms; repetition time = 2,000 ms; flip angle = 77°; field of view = 240 × 240 mm^2^; matrix = 64 × 64; slice thickness = 4 mm; slice gap = 0 mm; 38 interleaved axial slices.

### Structural Covariance Network Analysis

#### Deformation Based Morphometry

3D T1 images were preprocessed using Computational Anatomy Toolbox (CAT 12)^1^ running within Statistical Parametric Mapping (SPM 12)^2^. The preprocessing steps included denoising, correction for intensity inhomogeneity, and linear intensity scaling. Through utilizing the nonlinear transformation information of structural images from individual space to the standard Montreal Neurological Institute (MNI) space, a map recording the spatial deformation of each voxel was obtained from each subject. The deformation can be used as a means to quantify the map of local volume changes and be used to conduct statistical analysis directly (Chung et al., 2003). Finally, the DBM data were smoothed with a 6 mm full-width-half-maximum Gaussian kernel.

#### Independent Component Analysis

ICA is a method to decompose data into statistically independent components (ICs) without prior knowledge (Calhoun et al., 2001, 2003; Beckmann and Smith, 2004) which is used to obtain a spatially distinct large-scale structural covariance network (for further details see Xu et al., 2009). Smoothed DBM images were processed using the Group ICA of fMRI Toolbox (GIFT)^3^. Infomax algorithm was conducted, and the DBM images were decomposed into 20 spatial ICs based on group-level analysis (Bell and Sejnowski, 1995). A key issue with the ICA algorithm is that they are random and the results may be slightly different in different runs. ICASSO is a method for assessing the statistical reliability of estimated ICs (Himberg et al., 2004; Díez-Cirarda et al., 2018). We performed the infomax algorithm 20 times in ICASSO to ensure stability and validity. Finally, the IC maps were converted to z-statistic images and thresholded at a value of *z* > 3 (Beckmann and Smith, 2004; Xu et al., 2009). The network integrity score was calculated in a spatial regression against the 20 IC maps with the general linear model. This score represents the strength of structural covariance within the network (de Schipper et al., 2017). Thus, for each participant, one structural covariance network has one integrity score; and a high score means a strong structural covariance within the network.

Of note, to make sure the comparability between groups and evaluate the structural covariance network integrity difference, the ICA was conducted on all participants. Also, previous studies showed the structural covariance network was different between patients and controls (Alexander-Bloch et al., 2013). Therefore, we performed the same ICA procedures in PD patients and healthy controls, respectively to assess the morphological alteration of the structural covariance network in different groups. The spatial distribution of structural covariance networks in healthy controls was served as references. Of note, to illustrate the networks we identified were matched in two groups, dice were used and this part of the results was not used for further statistical analysis.

### Functional Network Analysis

Since decreased covariance between the anterior and posterior cingulate was found in PD patients, we specifically explored the change of functional connectivity between anterior and posterior cingulate subnetworks. It should be noted that there is no complete overlap between the functional network and structural covariance networks. Therefore, we conducted an independent ICA in rs-fMRI data and selected the corresponding functional network for further analysis.

#### Independent Component Analysis

Rs-fMRI data were preprocessed using the Data Processing and Analysis for Resting-State Brain Imaging (DPABI)^4^ according to the standard pipeline including the removal of the first 10 volumes, slice timing, realignment, spatial normalization and smoothing (6 × 6 × 6 mm^3^; Yan et al., 2016). ICA was conducted in smoothed fMRI data with the same procedures. Detail steps were described above.

#### Functional Network Selection

“Goodness-of-fit” (GOF) analysis was used to select the functional network that showed the best overlap with the altered structural covariance network. GOF was defined as the difference of the mean t-score of all voxels inside vs. outside the structural covariance network template (Seeley et al., 2009). This approach considers the connectivity strength when evaluating the spatial similarity. For every individual, each functional component had one GOF score. The higher the GOF score, the better the spatial overlap between the two networks. In the present study, the anterior and posterior cingulate structural covariance subnetworks obtained from healthy controls were used as the binary templates (threshold at a value of *z* > 3; Xu et al., 2009). One sample *T*-test was conducted to generate the t-maps of functional ICs in two groups, respectively. The functional ICs having the highest GOF score were selected as the anterior and posterior cingulate functional subnetworks.

#### Functional Network Connectivity

The functional connectivity between anterior and posterior cingulate subnetworks was assessed using the Functional Network Connectivity toolbox (FNC; Jafri et al., 2008; Wei et al., 2016)^5^. The time courses of the selected subnetworks were detrended, despiked, and filtered with a threshold at 0.013–0.24 Hz. Pearson’s correlation was conducted to evaluate the FNC between two subnetworks. Each subject had one FNC value. A higher FNC value represents stronger connectivity between the two subnetworks.

### Statistical Analysis

Statistical analysis was conducted using IBM SPSS Statistics 23.0 software. Independent samples *T*-test was used to evaluate the difference of the structural covariance network integrity and FNC value between two groups. Since age and sex had an impact on the integrity of the structural network (Montembeault et al., 2012; Spreng and Turner, 2013), they were regressed out as covariates. False discovery rate (FDR) correction was used to correct for multiple comparisons. The relationship between the integrity of the structural covariance network and FNC value was calculated using partial correlation analysis, adjusting the influences of age and sex.

In the longitudinal subgroup, the same procedures were performed before statistical analysis. A paired *T*-test was used to compare PD patients at baseline and follow-up. Independent samples *T*-test was used to compare controls and patients at baseline and follow-up accordingly.

## Results

### Demographic Characteristics

In the whole group (100 PD and 77 healthy controls), no significant difference was observed in age, sex, education, and MMSE score between patients and controls (Table 1). There is also no significant difference in age, sex, education, disease duration, UPDRS scores, PDQ-39 score, and MMSE score between PD patients in the whole group and the longitudinal subgroup (Table 2). This indicated that the two samples were well-matched in terms of clinical and demographic features. In a longitudinal subgroup, the mean time of follow-up in PD patients was 24 months. No significant difference was observed in age, sex, education, and MMSE score between controls and patients at baseline and follow-up (Table 3).

**Table 1:** Demographic characteristic.

	Controls (*n* = 77)	PD patients (*n* = 100)	*P*-value
Age	59.76 ± 7.63	59.77 ± 9.08	0.994
Sex (male/female)	30/47	55/45	0.121
Education	9.09 ± 3.57	8.15 ± 4.45	0.146
Duration	-	2.82 ± 1.74	-
H-Y stage	-	1.85 ± 0.64	-
UPDRS I	-	1.23 ± 1.45	-
UPDRS II	-	8.96 ± 5.21	-
UPDRS III	-	25.26 ± 22.27	-
UPDRS IV	-	0.96 ± 4.97	-
UPDRS total	-	36.05 ± 18.78	-
PDQ-39	-	24.18 ± 19.48	-
MMSE	27.93 ± 2.14	26.98 ± 3.42	0.053

*H-Y, Hoehn-Yahr; UPDRS, Unified Parkinson’s Disease Rating Scale; PDQ-39, Parkinson’s Disease Questionnaire (39 questions); MMSE, Mini-Mental State Examination*.

**Table 2:** Demographics characteristics between PD patients in cross-sectional and longitudinal cohorts.

	PD in the whole group (100)	PD in the longitudinal subgroup (51)	*P*-value
Age	59.77 ± 9.08	60.49 ± 8.19	0.635
Sex (male/female)	55/45	30/21	0.657
Education	8.15 ± 4.45	8.69 ± 4.46	0.490
Duration	2.82 ± 1.74	3.06 ± 2.00	0.441
H-Y stage	1.85 ± 0.64	1.96 ± 0.49	0.274
UPDRS I	1.23 ± 1.45	1.10 ± 1.14	0.571
UPDRS II	8.96 ± 5.21	8.18 ± 4.27	0.355
UPDRS III	25.26 ± 22.27	22.27 ± 12.03	0.200
UPDRS IV	0.96 ± 4.97	0.67 ± 1.34	0.680
UPDRS total	36.05 ± 18.78	32.41 ± 16.03	0.239
PDQ-39	24.18 ± 19.48	21.72 ± 17.57	0.453
MMSE	26.98 ± 3.42	27.75 ± 2.77	0.170

*H-Y, Hoehn-Yahr; UPDRS, Unified Parkinson’s Disease Rating Scale; PDQ-39, Parkinson’s Disease Questionnaire (39 questions); MMSE, Mini-Mental State Examination*.

**Table 3:** Demographics characteristics in the longitudinal subgroup.

	PD patients at baseline (51)	PD patients at follow-up (51)	Controls (70)	*P*-value			
				A	B	C	D
Age	60.49 ± 8.19	62.45 ± 8.11	59.76 ± 7.63	0.179	-	-	-
Sex (male/female)	30/21	30/21	30/47	0.121	-	-	-
Education	8.69 ± 4.46	8.69 ± 4.46	9.09 ± 3.57	0.451	-	-	-
Duration	3.06 ± 2.00	5.02 ± 1.92	-	-	-	-	-
H-Y stage	1.96 ± 0.49	2.15 ± 0.52	-	-	0.066	-	-
UPDRS I	1.10 ± 1.14	1.63 ± 1.95	-	-	0.097	-	-
UPDRS II	8.18 ± 4.27	7.78 ± 5.14	-	-	0.676	-	-
UPDRS III	22.27 ± 12.03	18.49 ± 10.48	-	-	0.007	-	-
UPDRS IV	0.67 ± 1.34	1.16 ± 1.29	-	-	0.062	-	-
UPDRS total	32.41 ± 16.03	29.06 ± 14.89	-	-	0.093	-	-
PDQ-39	21.72 ± 17.57	23.32 ± 20.68	-	-	0.678	-	-
MMSE	27.75 ± 2.77	27.45 ± 2.72	27.93 ± 2.14	0.588	-	-	-

*H-Y, Hoehn-Yahr; UPDRS, Unified Parkinson’s Disease Rating Scale; PDQ-39, Parkinson’s Disease Questionnaire (39 questions); MMSE, Mini-Mental State Examination; A, ANOVA; B, Paired *T*-test in PD patients at baseline and follow-up; C, Independent *T*-test between PD patients at baseline and controls; D, Independent *T*-test between PD patients at follow-up and controls*.

### Structural Covariance Network Alteration

**Figure 2:**
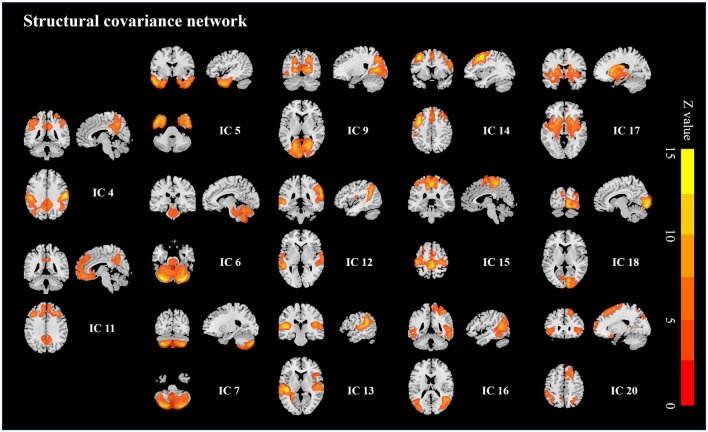
Structural covariance networks changes in PD patients. PD patients showed decreased integrity in the frontoparietal structural network and cingulate structural network (IC 4 and IC11).

Twenty ICs were obtained from all participants and six ICs composed of the non-brain tissues were excluded from the analysis. PD patients showed significantly decreased integrity in IC4 (*p* = 0.021) and IC11 (*p* = 0.009) compared to healthy controls while the resting 12 ICs showed no significant intergroup difference (Figure 2; FDR corrected).

**Figure 3:**
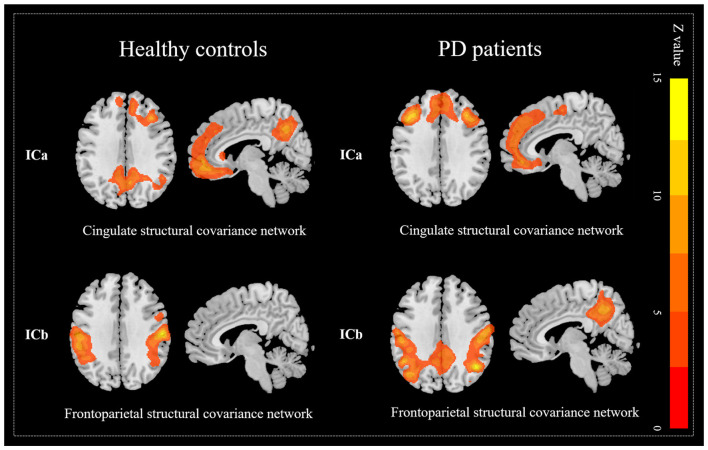
Decreased covariance between anterior and posterior cingulate structural subnetworks in PD patients. The posterior cingulate gyrus was not express in the cingulate network but expressed in the frontoparietal network which indicates the decreased covariance between anterior and posterior cingulate structural subnetworks.

Since the pattern of structural covariance network might be various from disease status, ICA was performed respectively to clarify the network patterns in each group. For the control group, 20 ICs were acquired and two of the ICs (ICa and ICb, Figure 3) were spatially overlapped with altered ICs mentioned above (Dice coefficient = 0.68). According to previous studies (Spreng and Turner, 2013; Hafkemeijer et al., 2014; de Schipper et al., 2017), ICa was defined as the cingulate structural covariance network which was mainly composed of anterior (anterior cingulate gyrus, superior and middle frontal cortices) and posterior (posterior cingulate gyrus, precuneus and angular) parts. It clued that the anterior and posterior parts of the cingulate area had a strong structural coupling. ICb was defined as the frontoparietal structural covariance network which was mainly composed of the precentral gyrus, postcentral gyrus, and inferior parietal gyrus. Similarly, for the PD patients, two ICs (ICa and ICb, Figure 3) were also spatially overlapped with the altered ICs obtained from all participants (Dice coefficient = 0.69). However, as is shown in Table 4 and Figure 3, the posterior cingulate gyri of PD patients were disappeared in the cingulate structural covariance network, which indicated the decreased structural covariance between anterior and posterior cingulate subnetworks.

**Table 4:** Altered structural networks in PD patients.

Networks Brain regions	Brain regions	Voxel size	MNI coordinates		
			*X*	*Y*	*Z*
**Healthy controls**					
**Cingulate network**
Anterior cingulate cortex and superior and middle frontal cortex		21,376	35	27	38
Posterior cingulate cortex, precuneus and angular		6,948	27	−53	50
**Frontoparietal network**					
Precentral, postcentral gyri and inferior parietal gyri		23,541	59	−18	30
**PD patients**					
**Cingulate network**					
Anterior cingulate cortex and superior and middle frontal cortex		29,015	−36	23	41
**Frontoparietal network**					
Precentral, postcentral gyri, inferior parietal gyri and angular		18,620	45	−59	33
Posterior cingulate cortex and precuneus		6,127	8	−53	41

*MNI, Montreal Neurological Institute*.

### Functional Network Connectivity Alteration

Based on the finding of structural dysconnectivity between anterior and posterior cingulate subnetworks in PD patients, we further explored the FNC change between anterior and posterior cingulate subnetworks.

**Figure 4:**
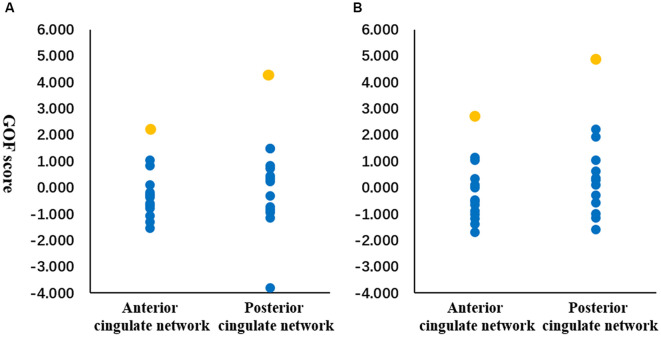
Goodness-of-fit (GOF) analysis between structural and functional networks. GOF was defined as the difference between the t-score mean within vs. outside structural network template. Orange dots represent the GOF score of the functional independent components (ICs) showed the highest similarity with the anterior and posterior cingulate structural subnetworks. Blue dots represent the GOF score of other functional ICs. GOF score was calculated in healthy controls **(A)** and patients **(B)**, respectively.

**Figure 5:**
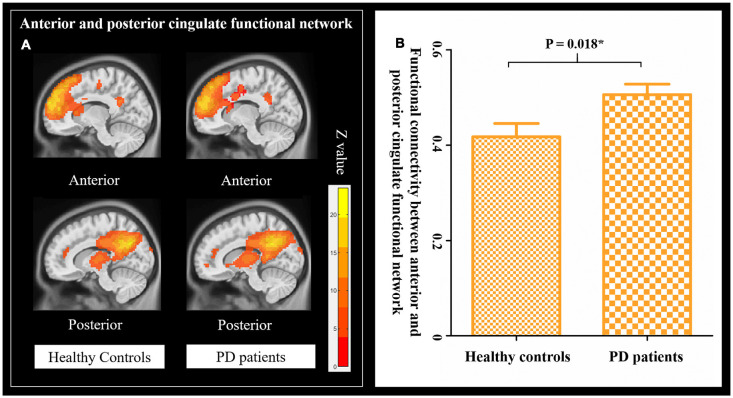
FNC in patients and healthy controls. **(A)** Anterior and posterior cingulate functional network in healthy controls and PD patients (Family wise error, FWE corrected, *p* = 0.001). **(B)** The changes of FNC between the anterior and posterior cingulate functional network in healthy controls and PD patients.

**Figure 6:**
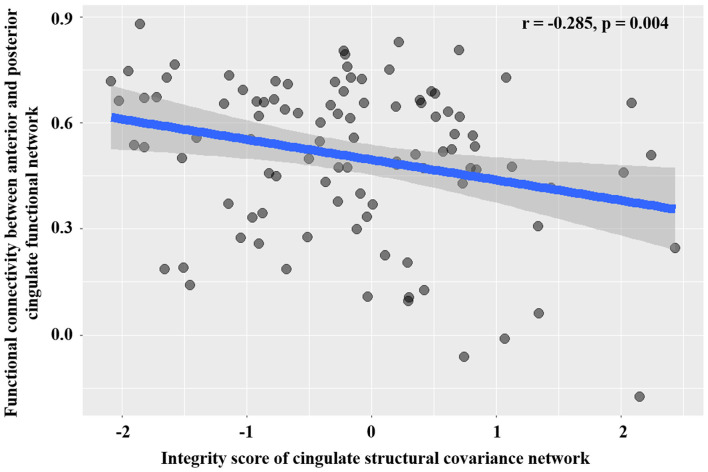
Association between structural network integrity and the strength of FNC.

By using the GOF analysis, the ICs showed the highest consistency with the anterior and posterior cingulate structural covariance networks that were selected as the corresponding functional networks (Figure 4). To visualize the selected anterior and posterior cingulate functional subnetworks, family-wise error (FWE, *p* < 0.001) correction was performed on the t-map of patients and controls (Figure 5A). PD showed increased FNC between anterior and posterior cingulate functional subnetworks (Figure 5B); and increased FNC was negatively correlated with decreased cingulate structural covariance network integrity in PD patients only (Figure 6).

### Longitudinal Analysis

**Figure 7:**
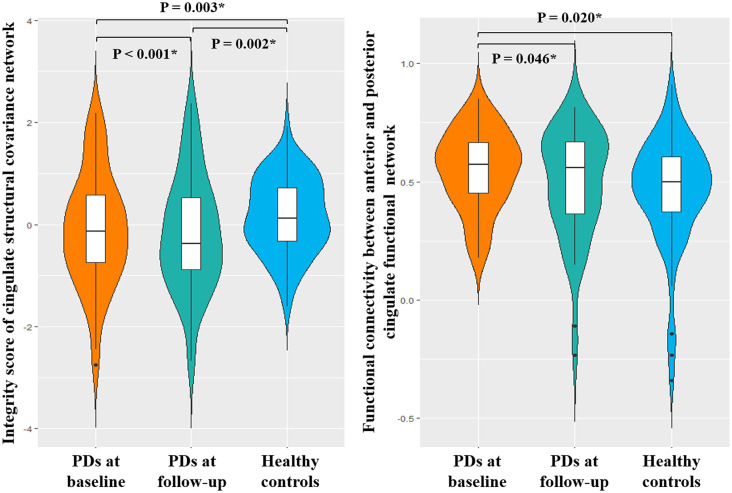
Longitudinal change of FNC.

In line with the cross-sectional analysis, the cingulate structural covariance network integrity was disrupted in PD patients at baseline and increasingly disrupted at follow-up. In functional network analysis, increased FNC was found in PD patients at baseline. The FNC was negatively correlated with the integrity of the cingulate structural covariance network in PD patients only (*r* = −0.238, *p* = 0.050). After an average of 24 months’ follow-up, the FNC between anterior and posterior cingulate subnetworks was decreased to the level of healthy controls (Figure 7). Also, the UPDRS I score was significantly correlated with the FNC between anterior and posterior cingulate subnetworks at follow-up (*r* = −0.385, *p* = 0.006). The FNC change was significantly correlated with the change of UPDRS I score (change = follow-up—baseline, *r* = −0.416, *P* = 0.003).

## Discussion

The present study provided insights regarding the structural covariance network alteration, and the association between structural covariance network and functional network in PD. (1) Significantly decreased integrity of the frontoparietal and cingulate structural covariance networks were found in PD patients. More importantly, structural covariance between anterior and posterior cingulate subnetworks was decreased in PD patients. (2) PD patients showed increased FNC between the anterior and posterior cingulate subnetworks at baseline, and the FNC was negatively correlated with the cingulate structural covariance network integrity. Longitudinally, the enhanced FNC at baseline was decreased after 24 months’ follow-up in PD patients.

### Structural Covariance Network Disruption

Network degeneration hypothesis points out that neurodegenerative disorders target specific large-scale networks (Seeley et al., 2009; Hafkemeijer et al., 2016). The frontoparietal structural covariance network mainly included the precentral, postcentral, and posterior parietal gyri. These brain regions are the primary area of the sensorimotor system and they work together to plan and execute movements. It is known that the typical clinical features of PD are motor dysfunction, and previous studies have reported that the atrophy in these regions was closely related to motor severity (Lyoo et al., 2011; Hwang et al., 2013; Sterling et al., 2017; Li et al., 2018). The cingulate structural covariance network mainly included the anterior cingulate gyrus, superior frontal cortex, middle frontal cortex, posterior cingulate cortex, precuneus, and angular gyrus. In line with previous studies (Seeley et al., 2009; Spreng and Turner, 2013; Hafkemeijer et al., 2014), this network was well-matched with the default mode network (DMN), which is closely related to cognition like memory and attention. Structural atrophy (Hwang et al., 2013; Xia et al., 2013; de Schipper et al., 2017) and functional dysconnectivity (Tessitore et al., 2012; Amboni et al., 2015; Hou et al., 2018; Schindlbeck and Eidelberg, 2018; Zhan et al., 2018) within this network were observed in PD patients, especially in patients with cognitive impairment. Moreover, the topography of the cingulate network resembled the PD-related cognitive pattern obtained from the metabolic imaging study (Tang et al., 2010). These results supported that the frontoparietal and cingulate structural covariance networks might be susceptible to PD related pathological substances.

Then, we further disclosed that the pattern of cingulate structural covariance network in PD patients was different from that in healthy controls. PD patients showed decreased covariance between anterior and posterior cingulate structural covariance subnetworks. It is known that the communication between anterior and posterior cingulate networks plays an essential role in information integration and closely associated with cognition (Delano-Wood et al., 2012; Metzler-Baddeley et al., 2012; Prell, 2018). The decreased covariance between them was possibly indicating the risk of transforming to cognitive impairment along the disease course. However, a preserved cognitive outcome was found in our group of patients. We assumed that functional compensation might play an important role in maintaining cognition. Congruent with our study, the loss of integrity in the cingulate structural covariance network was also reported in a recent study (de Schipper et al., 2017). This finding suggested that decreased covariance between anterior and posterior cingulate structural subnetworks was a hallmark of PD.

### Association Between Altered Structural Covariance Network and Functional Networks

PD patients showed increased FNC between anterior and posterior cingulate subnetwork which is spatial overlapped with the DMN (Beckmann et al., 2005; Damoiseaux et al., 2006). Increased FNC between anterior and posterior cingulate subnetworks was associated with decreased integrity of cingulate structural covariance network in PD patients. We postulated that increased FNC might represent a compensatory process for the disrupted cingulate structural covariance network, which might be important to maintain cognitive performance in PD patients (Hou et al., 2017; Prell, 2018). The functional connectivity of the DMN increases with the maturity of cognitive function; and the network activity decreases gradually with the decline of cognition in old age. Enhanced DMN activity in disease state might be an early adaptive response by increasing information integration and resource recruitment (Kehagia et al., 2013; Buckner and DiNicola, 2019). One study showed that PD patients present higher connectivity between PCC and an extensive cortex compared with healthy controls which might represent a compensatory process (Zhang et al., 2015). More importantly, increased functional connectivity in the PCC and middle prefrontal cortex which are the core regions of DMN was found in non-dementia PD patients, while PD patients with dementia showed significantly decreased functional connectivity (Chen et al., 2015; Gorges et al., 2015; Hou et al., 2017; Zhan et al., 2018). These researchers suggested that increased connectivity might be served as compensation for cognitive dysfunction in PD patients. Although the implication for compensatory changes is still unclear, evidence suggests that there are two main potential mechanisms contribute to compensation: PET studies suggesting that compensatory upregulation of D2-receptors and enzyme dopa decarboxylase were found in early untreated PD patients (Antonini et al., 1994; Kaasinen et al., 2000). Furthermore, the increased spatial extent of activation or increased connectivity patterns is also one of the mechanisms of compensation (Appel-Cresswell et al., 2010).

Moreover, our longitudinal observation showed significantly decreased FNC after 2 years in PD patients. Decreased FNC significantly correlated with the UPDRS I score which represents the cognition and emotion at follow-up and FNC change was significantly correlated with the change of UPDRS I score. These findings further indicated that the temporary increase in FNC might be a form of compensation; and this work provided a primary insight into the complex relationship between structural covariance network and functional network disruption in PD. However, direct evidence of compensation is still lacking and the mechanism of compensation should be interpreted cautiously.

The limitations of this study should be mentioned. Cognitive assessment is not extensive. No significant correlation was found between cognition and FNC or network integrity at baseline, we assume that this was caused by functional compensation. The sample size of longitudinal analysis was relatively small, the results should be interpreted cautiously. Future study with larger samples and longer time interval is needed to validate the current study.

## Conclusion

In conclusion, this study explained the complicated brain network modulation during the brain degeneration in PD. The frontoparietal and cingulate structural covariance networks were fragile in PD patients. And the disconnection of anterior and posterior of cingulate structural subnetworks might be compensated by a temporal enhanced functional connectivity.

## Data Availability Statement

The datasets generated for this study are available on request to the corresponding author.

## Ethics Statement

The studies involving human participants were reviewed and approved by the Medical Ethic Committee of the Second Affiliated Hospital of Zhejiang University School of Medicine. The patients/participants provided their written informed consent to participate in this study. Written informed consent was obtained from the individual(s) for the publication of any potentially identifiable images or data included in this article.

## Author Contributions

CZ and TGa were involved with study concept and design, acquisition of data, analysis, interpretation of data, and drafting/revising the manuscript. TGu, JinW, and XG were involved with the acquisition of data, analysis, and interpretation of data. WZ, PH, MX, QG, and XX were involved with data analysis and manuscript revision. SX, DK, and JiaW were responsible for manuscript revision. MZ was responsible for the study concept, revising the manuscript, obtaining funding, and study supervision. All authors read and approved the final manuscript.

## Conflict of Interest

The authors declare that the research was conducted in the absence of any commercial or financial relationships that could be construed as a potential conflict of interest.

## Footnotes

^1^http://dbm.neuro.uni-jena.de/cat12

^2^www.fil.ion.ucl.ac.uk/spm

^3^https://www.nitrc.org/projects/gift

^4^http://rfmri.org/dpabi

^5^http://mialab.mrn.org/software/fnc

## Abbreviations

PD: Parkinson’s disease
ICA: Independent component analysis
MRI: magnetic resonance imaging
DBM: Deformation-based morphometry
UPDRS: Unified Parkinson’s Disease Rating Scale
H-Y: Hoehn-Yahr
PDQ-39: Parkinson’s Disease Questionnaire (39 questions)
MMSE: Mini-Mental State Examination
MNI: Montreal Neurological Institute
GM: Gray matter
IC: Independent component
FNC: Functional network connectivity
GOF: Goodness-of-fit
FDR: False discovery rate
FEW: Family wise error
DMN: Default mode network.

## References

[B1] Antonini A. Schwarz J. Oertel W. H. Beer H. F. Madeja U. D. Leenders K. L. (1994). [^11^C]raclopride and positron emission tomography in previously untreated patients with Parkinson’s disease: influence of L-dopa and lisuride therapy on striatal dopamine D2-receptors. Neurology 44, 1325–1329. 10.1212/wnl.44.7.1325 8035939

[B2] Appel-Cresswell S. de la Fuente-Fernandez R. Galley S. McKeown M. J. (2010). Imaging of compensatory mechanisms in Parkinson’s disease. Curr. Opin. Neurol. 23, 407–412. 10.1097/WCO.0b013e32833b6019 20610991

[B3] Alexander-Bloch A. Giedd J. N. Bullmore E. (2013). Imaging structural co-variance between human brain regions. Nat. Rev. Neurosci. 14, 322–336. 10.1038/nrn3465 23531697 PMC4043276

[B4] Amboni M. Tessitore A. Esposito F. Santangelo G. Picillo M. Vitale C.. (2015). Resting-state functional connectivity associated with mild cognitive impairment in Parkinson’s disease. J. Neurol. 262, 425–434. 10.1007/s00415-014-7591-5 25428532

[B5] Baggio H. C. Segura B. Junque C. (2015). Resting-state functional brain networks in Parkinson’s disease. CNS Neurosci. Ther. 21, 793–801. 10.1111/cns.12417 26224057 PMC6093256

[B6] Beckmann C. F. DeLuca M. Devlin J. T. Smith S. M. (2005). Investigations into resting-state connectivity using independent component analysis. Philos. Trans. R. Soc. B Biol. Sci. 360, 1001–1013. 10.1098/rstb.2005.1634 16087444 PMC1854918

[B7] Beckmann C. F. Smith S. M. (2004). Probabilistic independent component analysis for functional magnetic resonance imaging. IEEE Trans. Med. Imaging 23, 137–152. 10.1109/tmi.2003.822821 14964560

[B8] Bell A. J. Sejnowski T. J. (1995). An information-maximization approach to blind separation and blind deconvolution. Neural Comput. 7, 1129–1159. 10.1162/neco.1995.7.6.1129 7584893

[B9] Braak H. Ghebremedhin E. Rub U. Bratzke H. Del Tredici K. (2004). Stages in the development of Parkinson’s disease-related pathology. Cell Tissue Res. 318, 121–134. 10.1007/s00441-004-0956-9 15338272

[B10] Buckner R. L. DiNicola L. M. (2019). The brain’s default network: updated anatomy, physiology and evolving insights. Nat. Rev. Neurosci. 20, 593–608. 10.1038/s41583-019-0212-7 31492945

[B11] Calhoun V. D. Adali T. Pekar J. J. Pearlson G. D. (2003). Latency (in)sensitive ICA. Group independent component analysis of fMRI data in the temporal frequency domain. NeuroImage 20, 1661–1669. 10.1016/S1053-8119(03)00411-7 14642476

[B12] Calhoun V. D. Adali T. Pearlson G. D. Pekar J. J. (2001). A method for making group inferences from functional MRI data using independent component analysis. Hum. Brain Mapp. 14, 140–151. 10.1002/hbm.1048 11559959 PMC6871952

[B13] Chen B. Fan G. G. Liu H. Wang S. (2015). Changes in anatomical and functional connectivity of Parkinson’s disease patients according to cognitive status. Eur. J. Radiol. 84, 1318–1324. 10.1016/j.ejrad.2015.04.014 25963506

[B14] Chung M. K. Worsley K. J. Robbins S. Paus T. Taylor J. Giedd J. N.. (2003). Deformation-based surface morphometry applied to gray matter deformation. NeuroImage 18, 198–213. 10.1016/s1053-8119(02)00017-4 12595176

[B15] Delano-Wood L. Stricker N. H. Sorg S. F. Nation D. A. Jak A. J. Woods S. P.. (2012). Posterior cingulum white matter disruption and its associations with verbal memory and stroke risk in mild cognitive impairment. J. Alzheimers Dis. 29, 589–603. 10.3233/jad-2012-102103 22466061 PMC3341099

[B16] de Schipper L. J. Hafkemeijer A. van der Grond J. Marinus J. Henselmans J. M. L. van Hilten J. J. (2018). Altered whole-brain and network-based functional connectivity in Parkinson’s disease. Front. Neurol. 9:419. 10.3389/fneur.2018.00419 29928255 PMC5997827

[B17] de Schipper L. J. van der Grond J. Marinus J. Henselmans J. M. L. van Hilten J. J. (2017). Loss of integrity and atrophy in cingulate structural covariance networks in Parkinson’s disease. NeuroImage Clin. 15, 587–593. 10.1016/j.nicl.2017.05.012 28652971 PMC5477092

[B18] Damoiseaux J. S. Rombouts S. A. Barkhof F. Scheltens P. Stam C. J. Smith S. M.. (2006). Consistent resting-state networks across healthy subjects. Proc. Natl. Acad. Sci. U S A 103, 13848–13853. 10.1073/pnas.0601417103 16945915 PMC1564249

[B19] Díez-Cirarda M. Ibarretxe-Bilbao N. Peña J. Ojeda N. (2018). Neurorehabilitation in Parkinson’s disease: a critical review of cognitive rehabilitation effects on cognition and brain. Neural Plast. 2018:2651918. 10.1155/2018/2651918 29853840 PMC5960507

[B20] Gottlich M. Munte T. F. Heldmann M. Kasten M. Hagenah J. Kramer U. M. (2013). Altered resting state brain networks in Parkinson’s disease. PLoS One 8:e77336. 10.1371/journal.pone.0077336 24204812 PMC3810472

[B21] Gorges M. Müller H. P. Lulé D. Pinkhardt E. H. Ludolph A. C. Kassubek J. (2015). To rise and to fall: functional connectivity in cognitively normal and cognitively impaired patients with Parkinson’s disease. Neurobiol. Aging 36, 1727–1735. 10.1016/j.neurobiolaging.2014.12.026 25623332

[B22] Hafkemeijer A. Altmann-Schneider I. de Craen A. J. Slagboom P. E. van der Grond J. Rombouts S. A. (2014). Associations between age and gray matter volume in anatomical brain networks in middle-aged to older adults. Aging Cell 13, 1068–1074. 10.1111/acel.12271 25257192 PMC4326918

[B23] Hafkemeijer A. Möller C. Dopper E. G. P. Jiskoot L. C. van den Berg-Huysmans A. A. van Swieten J. C.. (2016). Differences in structural covariance brain networks between behavioral variant frontotemporal dementia and Alzheimer’s disease. Hum. Brain Mapp. 37, 978–988. 10.1002/hbm.23081 26660857 PMC6867562

[B24] Himberg J. Hyvarinen A. Esposito F. (2004). Validating the independent components of neuroimaging time series *via* clustering and visualization. NeuroImage 22, 1214–1222. 10.1016/s1053-8119(04)00166-1 15219593

[B26] Hou Y. Luo C. Yang J. Ou R. Liu W. Song W.. (2017). Default-mode network connectivity in cognitively unimpaired drug-naive patients with rigidity-dominant Parkinson’s disease. J. Neurol. 264, 152–160. 10.1007/s00415-016-8331-9 27848084

[B25] Hou Y. Yang J. Luo C. Ou R. Zou Y. Song W.. (2018). Resting-state network connectivity in cognitively unimpaired drug-naïve patients with rigidity-dominant Parkinson’s disease. J. Neurol. Sci. 395, 147–152. 10.1016/j.jns.2018.10.003 30321795

[B27] Hughes A. J. Daniel S. E. Kilford L. Lees A. J. (1992). Accuracy of clinical diagnosis of idiopathic Parkinson’s disease: a clinico-pathological study of 100 cases. J. Neurol. Neurosurg. Psychiatry 55, 181–184. 10.1136/jnnp.55.3.181 1564476 PMC1014720

[B28] Hwang K. S. Beyer M. K. Green A. E. Chung C. Thompson P. M. Janvin C.. (2013). Mapping cortical atrophy in Parkinson’s disease patients with dementia. J. Parkinsons Dis. 3, 69–76. 10.3233/JPD-120151 23938313 PMC4018208

[B29] Jafri M. J. Pearlson G. D. Stevens M. Calhoun V. D. (2008). A method for functional network connectivity among spatially independent resting-state components in schizophrenia. NeuroImage 39, 1666–1681. 10.1016/j.neuroimage.2007.11.001 18082428 PMC3164840

[B30] Jellinger K. A. (2012). Neuropathology of sporadic Parkinson’s disease: evaluation and changes of concepts. Mov. Disord. 27, 8–30. 10.1002/mds.23795 22081500

[B31] Kaasinen V. Ruottinen H. M. Nagren K. Lehikoinen P. Oikonen V. Rinne J. O. (2000). Upregulation of putaminal dopamine D2 receptors in early Parkinson’s disease: a comparative PET study with [^11^C] raclopride and [^11^C]N-methylspiperone. J. Nucl. Med. 41, 65–70. 10647606

[B32] Kehagia A. A. Barker R. A. Robbins T. W. (2013). Cognitive impairment in Parkinson’s disease: the dual syndrome hypothesis. Neurodegener. Dis. 11, 79–92. 10.1159/000341998 23038420 PMC5079071

[B33] Lee P.-L. Chou K.-H. Lu C.-H. Chen H.-L. Tsai N.-W. Hsu A.-L.. (2018). Extraction of large-scale structural covariance networks from grey matter volume for Parkinson’s disease classification. Eur. Radiol. 28, 3296–3305. 10.1007/s00330-018-5342-1 29532237

[B34] Li X. Xing Y. Martin-Bastida A. Piccini P. Auer D. P. (2018). Patterns of grey matter loss associated with motor subscores in early Parkinson’s disease. NeuroImage Clin. 17, 498–504. 10.1016/j.nicl.2017.11.009 29201638 PMC5700824

[B35] Lyoo C. H. Ryu Y. H. Lee M. S. (2011). Cerebral cortical areas in which thickness correlates with severity of motor deficits of Parkinson’s disease. J. Neurol. 258, 1871–1876. 10.1007/s00415-011-6045-6 21512741

[B36] Mak E. Su L. Williams G. B. Firbank M. J. Lawson R. A. Yarnall A. J.. (2015). Baseline and longitudinal grey matter changes in newly diagnosed Parkinson’s disease: ICICLE-PD study. Brain 138, 2974–2986. 10.1093/brain/awv211 26173861 PMC4671477

[B37] Metzler-Baddeley C. Jones D. K. Steventon J. Westacott L. Aggleton J. P. O’Sullivan M. J. (2012). Cingulum microstructure predicts cognitive control in older age and mild cognitive impairment. J. Neurosci. 32, 17612–17619. 10.1523/JNEUROSCI.3299-12.2012 23223284 PMC6621654

[B38] Montembeault M. Joubert S. Doyon J. Carrier J. Gagnon J. F. Monchi O.. (2012). The impact of aging on gray matter structural covariance networks. NeuroImage 63, 754–759. 10.1016/j.neuroimage.2012.06.052 22776455

[B39] Pan P. L. Song W. Shang H. F. (2012). Voxel-wise meta-analysis of gray matter abnormalities in idiopathic Parkinson’s disease. Eur. J. Neurol. 19, 199–206. 10.1111/j.1468-1331.2011.03474.x 21762435

[B40] Prell T. (2018). Structural and functional brain patterns of non-motor syndromes in Parkinson’s disease. Front. Neurol. 9:138. 10.3389/fneur.2018.00138 29593637 PMC5858029

[B42] Schindlbeck K. A. Eidelberg D. (2018). Network imaging biomarkers: insights and clinical applications in Parkinson’s disease. Lancet Neurol. 17, 629–640. 10.1016/s1474-4422(18)30169-8 29914708

[B43] Seeley W. W. Crawford R. K. Zhou J. Miller B. L. Greicius M. D. (2009). Neurodegenerative diseases target large-scale human brain networks. Neuron 62, 42–52. 10.1016/j.neuron.2009.03.024 19376066 PMC2691647

[B44] Spreng R. N. Turner G. R. (2013). Structural covariance of the default network in healthy and pathological aging. J. Neurosci. 33, 15226–15234. 10.1523/JNEUROSCI.2261-13.2013 24048852 PMC3776065

[B45] Sterling N. W. Du G. Lewis M. M. Swavely S. Kong L. Styner M.. (2017). Cortical gray and subcortical white matter associations in Parkinson’s disease. Neurobiol. Aging 49, 100–108. 10.1016/j.neurobiolaging.2016.09.015 27776262 PMC5154847

[B46] Tang C. C. Poston K. L. Eckert T. Feigin A. Frucht S. Gudesblatt M.. (2010). Differential diagnosis of parkinsonism: a metabolic imaging study using pattern analysis. Lancet Neurol. 9, 149–158. 10.1016/S1474-4422(10)70002-8 20061183 PMC4617666

[B47] Tessitore A. Esposito F. Vitale C. Santangelo G. Amboni M. Russo A.. (2012). Default-mode network connectivity in cognitively unimpaired patients with Parkinson disease. Neurology 79, 2226–2232. 10.1212/WNL.0b013e31827689d6 23100395

[B48] Wei L. Q. Chen Q. Tang L. L. Zhuang C. Zhu W. R. Lin N. (2016). A porous metal-organic framework with a unique hendecahedron-shaped cage: structure and controlled drug release. Dalton Trans. 45, 3694–3697. 10.1039/c5dt04379d 26842630

[B49] Xia J. Miu J. Ding H. Wang X. Chen H. Wang J.. (2013). Changes of brain gray matter structure in Parkinson’s disease patients with dementia. Neural Regen. Res. 8, 1276–1285. 10.3969/j.issn.1673-5374.2013.14.004 25206422 PMC4107646

[B50] Xu L. Groth K. M. Pearlson G. Schretlen D. J. Calhoun V. D. (2009). Source-based morphometry: The use of independent component analysis to identify gray matter differences with application to schizophrenia. Hum. Brain Mapp. 30, 711–724. 10.1002/hbm.20540 18266214 PMC2751641

[B51] Yan C. G. Wang X. D. Zuo X. N. Zang Y. F. (2016). DPABI: data processing and analysis for (resting-state) brain imaging. Neuroinformatics 14, 339–351. 10.1007/s12021-016-9299-4 27075850

[B52] Zeighami Y. Ulla M. Iturria-Medina Y. Dadar M. Zhang Y. Larcher K. M.. (2015). Network structure of brain atrophy in de novo Parkinson’s disease. Elife 4:e08440. 10.7554/eLife.08440 26344547 PMC4596689

[B53] Zhang J. Bi W. Zhang Y. Zhu M. Zhang Y. Feng H.. (2015). Abnormal functional connectivity density in Parkinson’s disease. Behav. Brain Res. 280, 113–118. 10.1016/j.bbr.2014.12.007 25496782

[B54] Zhan Z. W. Lin L. Z. Yu E. H. Xin J. W. Lin L. Lin H. L.. (2018). Abnormal resting-state functional connectivity in posterior cingulate cortex of Parkinson’s disease with mild cognitive impairment and dementia. CNS Neurosci. Ther. 24, 897–905. 10.1111/cns.12838 29500931 PMC6490031

